# Merging Resource Availability with Isotope Mixing Models: The Role of Neutral Interaction Assumptions

**DOI:** 10.1371/journal.pone.0022015

**Published:** 2011-07-07

**Authors:** Justin D. Yeakel, Mark Novak, Paulo R. Guimarães, Nathaniel J. Dominy, Paul L. Koch, Eric J. Ward, Jonathan W. Moore, Brice X. Semmens

**Affiliations:** 1 Department of Ecology and Evolutionary Biology, University of California Santa Cruz, Santa Cruz, California, United States of America; 2 Departamento de Ecologia, Instituto de Biociências, Universidade de São Paulo, São Paulo, São Paulo, Brazil; 3 Department of Anthropology & Department of Biological Sciences, Dartmouth College, Hanover, New Hampshire, United States of America; 4 Department of Earth and Planetary Sciences, University of California Santa Cruz, Santa Cruz, California, United States of America; 5 Northwest Fisheries Science Center, National Marine Fisheries Service, National Oceanic and Atmospheric Administration, Seattle, Washington, United States of America; 6 Department of Biological Sciences, Simon Fraser University, Burnaby, British Columbia, Canada; National Institute of Water & Atmospheric Research, New Zealand

## Abstract

**Background:**

Bayesian mixing models have allowed for the inclusion of uncertainty and prior information in the analysis of trophic interactions using stable isotopes. Formulating prior distributions is relatively straightforward when incorporating dietary data. However, the use of data that are related, but not directly proportional, to diet (such as prey availability data) is often problematic because such information is not necessarily predictive of diet, and the information required to build a reliable prior distribution for all prey species is often unavailable. Omitting prey availability data impacts the estimation of a predator's diet and introduces the strong assumption of consumer ultrageneralism (where all prey are consumed in equal proportions), particularly when multiple prey have similar isotope values.

**Methodology:**

We develop a procedure to incorporate prey availability data into Bayesian mixing models conditional on the similarity of isotope values between two prey. If a pair of prey have similar isotope values (resulting in highly uncertain mixing model results), our model increases the weight of availability data in estimating the contribution of prey to a predator's diet. We test the utility of this method in an intertidal community against independently measured feeding rates.

**Conclusions:**

Our results indicate that our weighting procedure increases the accuracy by which consumer diets can be inferred in situations where multiple prey have similar isotope values. This suggests that the exchange of formalism for predictive power is merited, particularly when the relationship between prey availability and a predator's diet cannot be assumed for all species in a system.

## Introduction

### Background

Trophic interactions are fundamental components of biodiversity, directly contributing to ecosystem organization and dynamics [1]. Accurate reconstruction and quantification of the strengths of trophic interactions remains a challenge in most ecological systems [2]. Often, it is impossible to directly observe or document feeding relationships [3]. In many such cases, ratios of stable isotopes (typically those of carbon: ^13^C∶^12^C and nitrogen: ^15^N∶^14^N) can be used to investigate the diets of consumers [4]. Carbon isotope ratios distinguish primary producers that use different photosynthetic pathways (or that differ in other physiological or physiognomic attributes) and are conserved in the tissues of consumers, whereas ratios of nitrogen isotopes are strongly sensitive to trophic level (though they may vary spatially or with primary producer functional type). Accordingly, stable isotope data of both consumer and potential prey can be used to quantify a consumer's resource niche, can provide dietary information relevant across a range of temporal and spatial scales, and can distinguish dietary differences across hierarchies of animal communities [5], [6], [7], [8].

The extent to which inference can be successfully drawn from stable isotope data is dependent upon the isotopic uniqueness of a consumer's prey (here ‘prey’ may refer to any resource that is consumed by an organism). When different potential prey are isotopically distinct and the number of prey are greater than the number of isotopic tracers +1, analytical mixing models may be employed to assess the possible contributions of prey to a consumer's diet [9], [10], [11], [12]. Because many different combinations of dietary sources can produce a given set of isotope values under these conditions, quantitative tools are required to parse less likely trophic interactions from those that are more likely to occur. Bayesian mixing models can be used to numerically simulate posterior probability distributions that quantify the range and likelihood of all potential source combinations [7], [13], [14]. This approach requires prior knowledge of a consumer's diet to be designated, even if that knowledge is specified to be uninformative (i.e. that each prey item has an equal probability of contributing to the predator's diet). In addition, all isotopic mixing models require the accurate specification of prey isotope values and trophic fractionation factors [15].

Despite recent advances in Bayesian mixing models, estimates of dietary relationships may be inaccurate or uninformative when multiple prey are isotopically similar (this issue is problematic in non-Bayesian mixing models as well) [11]. Here we address the issue of isotopic similarity among prey that are not equally available to a consumer. Because the relative availability of a specific prey will theoretically have a direct impact on the consumer's diet, we propose a method to employ data reflecting these differences in availability to weight the probability distributions of a consumer's reliance on a particular prey. Because the precise relationship between a prey's inclusion to a predator's diet and a prey's availability is uncertain (and is not necessarily applicable to all prey in a predator's diet), we condition the influence of availability data on the isotopic uniqueness of a given prey.

### Incorporating Biological Information

The quantification of a consumer's diet implicitly assumes that all dietary resources included in an analysis are potentially important. If an uninformative prior is used in a Bayesian mixing model, all prey are assumed to contribute equally to a predator's diet *a priori*. This specification allows isotopic data to maximally influence the results of the mixing model. Thus, in a system with *n* potential prey sources, the *a priori* assumption is that the consumer is an ultrageneralist, consuming a unit of biomass of prey *i* with the probability: *c_i_* = *n^−1^*. The unique isotopic distribution of prey relative to the consumer's isotope values can modify this probability. Nevertheless, the *a priori* assumption will be especially relevant when two prey occupy the same isotopic space. Here, the model will predict that isotopically similar prey contribute equally to a consumer's diet.

Additional biological information regarding prey sources may render such generalizations over-simplistic; if the relative availability of prey on the landscape is known (*α_1_, α_2_, … ,α_n_*; where the sum of these elements is equal to unity), the underlying assumption may be revised to reflect these differences such that the probability of consuming a prey source is *c_i_* = *α_i_*. Accordingly, the system operates under conditions imposed by interaction neutrality (consumption in proportion to prey abundance) [16], [17], as opposed to ultrageneralism (consumption in proportion to the number of prey available to the consumer).

In theory, prey availability data, as quantified by abundance, biomass, consumer preference, or a combination thereof, can be used to inform dietary relationships within the framework of traditional Bayesian mixing models. We acknowledge upfront that, like all aspects of isotopic mixing models, such a practice should be used with caution. Specifically, there exists an implicit assumption that prey availability and consumer-prey interactions are directly related for every prey item that is included in the analysis. Importantly, the calculation of a consumer's diet based on prey availability and/or isotopic data are not equivalently informative. Isotopic data of a consumer and its potential prey are empirical measurements that track the flow of matter within a physical system. The relationship between isotopic data and trophic interactions has been well established in many ecosystems [4], [6], [18], [19]. On the other hand, the relationship between prey availability and diet choice is still not well understood [20], need not be system-specific [21], and may differ across prey, even for the same predator [21]. In these scenarios, prey availability data should not be introduced indiscriminately to inform Bayesian mixing models. Additionally, the use of prior information in Bayesian mixing models is subject to a number of important constraints, such that incorporation of biological information by means other than the Bayesian paradigm may be advantageous, depending on what data are available for a particular system.

### Post-hoc adjustments vs. the formulation of Prior Distributions

Bayesian isotope mixing models allow researchers to apply prior information to an isotopic system, thereby making use of different sources of biological data that provide information regarding a consumer's diet. Prior distributions represent the *a priori* knowledge of a consumer-resource relationship, and can be parameterized by direct or indirect measurements of prey consumption by a predator. The effect that a prior distribution has, relative to the likelihood, is partially dependent upon the uncertainty, or variance, of the prior distribution [22]. The formulation of informative priors is based on two logical assumptions. First, the Bayesian algorithm assumes that the data used to formulate the prior and likelihood are determinants of the same variable (in this case, diet). Second, the parameterization of prior probability distributions is contingent on calculable metrics, such as the mean and variance.

There are scenarios where one or both of these assumptions cannot be met. Regarding the first assumption, an example involving the use of gut contents data to formulate a prior for an isotope mixing model is presented in Moore and Semmens [13]. Because both gut contents data and the isotope values of a system are indirect measures of diet (*θ*), they lend themselves to a Bayesian framework wherein the posterior probability *p*(*θ*|*y*) is informed by both the prior *p*(*θ*) and the likelihood *p*(*y*|*θ*)
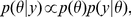
(1)where *y* represents isotopic data, and *θ* is a dietary parameter. According to Eq. (1), it is evident that *p*(*θ*|*y*) and *p*(*θ*) must both describe diet. Alternatively, if *p*(*θ*) represents measures of prey availability to a consumer and *p*(*y*|*θ*) is the likelihood shaped by isotopic (dietary) data, it is unclear what *p*(*θ*|*y*) describes, *unless* it is assumed that availability is directly correlated with diet. Prey availability would be an indirect measure of diet if species interactions follow neutral dynamics [16], [17]. For predator-prey interactions that are influenced by non-neutral, prey-specific ecological factors such as predator preference, prey defense, or habitat variability, measurements of prey availability cannot lend themselves to indiscriminant use as prior distributions as they no longer have a one-to-one relationship with their contribution to a consumer's diet.

As importantly, the second assumption requires that prior distribution parameters be calculable. Estimates of both mean and variance must be made for all prey species in the predator's diet to formulate appropriate priors. In systems where isotopic data are likely to be of maximum utility (e.g. systems that are difficult to observe directly), such parameters are often difficult (if not impossible) to estimate well, rendering prey availability data to parameterize prior distributions unviable. Moreover, in systems where few measurements of prey availability exist, or when count (census) data are used, variance is often underestimated [23], which can result in inaccurate posterior distributions [24]. Often, prey availability is better characterized for some species than for others. Differences in body size, temporal habitat use, and many other factors may confound parameterization of availability distributions of some species, even if the same quantities are well known for others. This renders the formulation of an accurate prior distribution difficult or impossible. In order to avoid these pitfalls, and to maximize the utility of isotope mixing models, a more targeted procedure designed to inform isotope-based measurements of a consumer's diet in a post-hoc fashion is warranted.

Here we introduce a procedure that incorporates, and appropriately weights, biologically relevant information into the posterior distributions of a Bayesian isotope mixing model. With this approach, the assumption of interaction neutrality is incorporated into mixing model results conditional on the isotopic similarity of prey sources. This condition preserves the intrinsic differences between direct measurements that describe a system (isotopic data), and independent data that may be theoretically linked to a system (prey availability data). First, we introduce the utility of this approach with a hypothetical isotopic system. We then test the effectiveness of our approach at increasing the accuracy of trophic interaction estimates by comparing isotope data and independently estimated foraging rates of a whelk predator feeding on its prey in a New Zealand intertidal community.

## Methods

### Weighting of Bayesian isotope mixing models

Bayesian isotope mixing model output is often summarized in a matrix of estimated dietary contribution vectors (ρ*_i,…,n_*; accepted results from a Markov Chain Monte Carlo, MCMC, or a Sampling-Importance-Resampling, SIR, algorithm), where each element in the vector is the contribution of a potential prey source, and each proposed vector sums to unity [13]. Proportional prey availability can be incorporated into the vector that defines the contribution of dietary sources for a particular consumer by multiplication and renormalization. Because direct physical measurements of trophic relationships (e.g. isotopic data) are likely to be more informative than the idealized assumption of a relationship between prey availability and diet, we allow prey availability data to influence dietary contribution results only in proportion to the extent that the prey have similar isotope values. As such, our model allows availability data to influence the source contribution vector in proportion to the pairwise isotopic overlap between prey. Isotopic overlap is proportional to the probability that the isotopic values of two sources are misidentified. Relative to a single pair of potential prey within a larger system, we define the impact of prey availability and isotopic data on final contribution-to-diet values to be exactly inversely proportional: when there is no overlap of prey sources, isotopic data are singularly informative; when there is complete overlap of prey sources, availability data are singularly informative (for the prey pair in question). We utilize Pianka's measure of density overlap to estimate the degree of isotopic overlap between two prey sources with normally distributed isotope values. Pianka's measure of overlap (*w_ij_*) is defined by
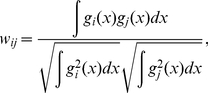
(2)where *g_i,j_(x)* represent multivariate normal distributions of isotope values for overlapping prey sources *i* and *j* [25]. Solving Eq. (2) with the assumption of bivariate normality yields
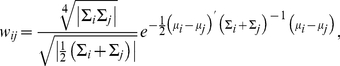
(3)where *μ_i_* and *μ_j_* are vectors of bivariate distribution means, Σ*_i_* and Σ*_j_* are covariance matrices of prey *i* and *j*, respectively, and 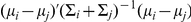 is the square of the Mahalanobis Distance (a generalization of Euclidean Distance) [26]. While this metric is capable of handling isotopic distributions with alternative non-Gaussian covariance structures, mixing models have not yet taken isotopic covariance into account, and we will not discuss it further. The application of an alternative measure of overlap, Morisita's metric [27], provided nearly identical results (not reported).

As noted above, isotopic overlap represents the probability of mistaking one prey source for another; in systems where isotope values for prey *i* and *j* are completely distinct (*w_ij_* = 0), assumptions of consumption based on prey availability have no influence, whereas isotopic data are regarded as singularly effective for determining a consumer's likely diet. If two prey have similar isotope values (0<*w_ij_*≤1), prey availability data are incorporated, in proportion to *w_ij_*, to estimate the final contribution-to-diet values. Accordingly, prey availability data are weighted to become increasingly informative as *w_ij_* increases. If *w_ij_* = 1 (exact isotopic overlap between two prey), isotopic and prey availability data are equally informative. We define the strength of this weighting value for prey *i* and *j* as
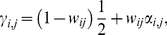
(4)where *w_ij_* is the degree of isotopic overlap (Eqns. 2,3), and *α_i,j_* are proportional availability measurements of prey *i* and *j* (for example, if prey *i* and *j* had proportional biomass measurements of 0.25 and 0.75, respectively, then *α_i_* = 0.25 and *α_j_* = 0.75). Therefore, the final weighted mixing model output is calculated as
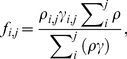
(5)where *ρ_i,j_* represents a vector of estimated contribution-to-diet values for prey *i* and *j* from a given iteration of the MCMC or SIR algorithm in a Bayesian isotope mixing model without including knowledge of relative availability. This process is employed iteratively across all proposed mixing model contribution-to-diet vectors, resulting in final probability distributions that are skewed towards relative availability (*α_i_*, *α_j_*) for prey *i* and *j* in proportion to their isotopic similarity. As an alternative to a single ratio of two integers (e.g. *α_i_* = 0.25, *α_j_* = 0.75), relative availability can be incorporated as a probability distribution. This ability is excluded from these analyses for simplicity.

### Application to a New Zealand intertidal food web

To assess the empirical power of our method, we applied mixing models with and without our weighting procedure to stable isotope data obtained from a predator-prey system of the New Zealand intertidal [28], [29]. Model results were then compared to biomass-weighted feeding rates calculated from independent observational data.

The focal predator is the gastropod whelk, *Haustrum* ( = *Lepsiella*) *scobina*, which is common along the rocky intertidal shores of New Zealand. We established the diet of *H. scobina* at a focal study site (Tauranga Head, 41°46′26″S, 171°27′20″E) by performing systematic low-tide feeding surveys of the population in pre-determined mid- and high-intertidal areas of the shore. Each encountered whelk was counted and carefully examined to determine feeding activity. The sizes of all whelks and their identified prey items were measured to within ±1 mm [30]. In total, 2668 whelks where encountered in 20 surveys performed over a 2-year period (May 2005–July 2007). On average, 20.8% of all individuals were actively feeding such that a total of 554 feeding observations on 8 prey species were recorded. The eight observed prey species were: the snails, *Austrolittorina antipodum (Aa)*, *A. cincta (Ac)*, and *Risellopsis varia (Rv)*; a limpet *Notoacmea* sp. (*Nr*); the mussels *Mytilus galloprovincialis* (*Mg*) and *Xenostrobus pulex (Xp)*; and the barnacles *Chamaesipho columna* (*Cc*) and *Epopella plicata (Ep)* (Table 1). The saturated nature of the resultant species accumulation curve suggests that most of *H. scobina*'s prey were documented (Fig. S1). Further details are provided in Novak [30].

**Table 1:** *Haustrum scobina* prey-specific foraging metrics.

Prey	Abb.	Feeding obser.	Density (m^−2^)	Handling time (days)	Per capita attack rate^1^	Feeding rate^2^	Proportional diet contribution
**Snails and limpets**							
*Austrolittorina antipodum*	Aa	4	159.8	0.6	2.03×10^−5^	1.84×10^−6^	2.76×10^−3^
*Austrolittorina cincta*	Ac	3	1921.6	0.4	2.02×10^−6^	5.34×10^−7^	8.01×10^−4^
*Notoacmea sp.*	Nr	1	213.1	0.2	1.04×10^−5^	1.66×10^−6^	2.49×10^−3^
*Risellopsis varia*	Rv	2	163.9	0.6	1.01×10^−5^	5.35×10^−7^	8.02×10^−4^
**Mussels**							
*Mytilus galloprovincialis*	Mg	5	222.2	2.0	5.21×10^−6^	7.47×10^−6^	1.12×10^−2^
*Xenostrobus pulex*	Xp	213	4771.4	1.3	1.59×10^−5^	2.73×10^−4^	4.10×10^−1^
**Barnacles**							
*Chamaesipho columna*	Cc	321	84445.0	0.6	3.05×10^−6^	3.50×10^−4^	5.26×10^−1^
*Epopella plicata*	Ep	5	1028.1	1.0	2.28×10^−6^	3.10×10^−5^	4.65×10^−2^

1 Number of prey eaten per whelk per prey available per m^2^ per day.

2 Dry tissue grams of prey eaten per whelk per m^2^ per day.

#### Observational estimates of prey contributions

We used the observational method of Novak and Wootton [29] to convert observed frequencies of predation events to prey-specific estimates of *H. scobina*'s feeding rates. This method required an estimation of mean prey-specific abundances and the mean prey-specific handling time required by *H. scobina* individuals to consume a prey item. Species' densities were estimated using 10–15 quadrats measuring 0.25 m^2^, randomly distributed across three 20-m transects positioned within each mid- and high-intertidal zone. Abundance surveys were repeated three times (May–July 2005, January–February 2006, May 2006) such that site-wide mean prey densities were estimated on the basis of 60–90 quadrats (Table 1). *H. scobina*'s handling times (the time required by an individual to drill and ingest a prey item) were measured in controlled laboratory experiments where prey identity and relative predator-prey body size could be manipulated independently (n = 208). The prey-specific relationships observed between these variables allowed us to estimate the expected handling time of each feeding event observed in the field. These data were then used to calculate prey-specific mean handling times (Table 1). Further details are provided in Novak [30].

Data from the feeding surveys, abundance surveys, and field-estimated handling-times were combined to calculate prey-specific per capita attack rates (*c_i_*, the number of prey eaten per predator per prey available per m^2^ per day) as
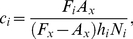
(6)where *h_i_* and *N_i_* respectively denote the *i* ^th^ prey's mean handling time (days) and mean density (#⋅m^−2^), *A_x_* denotes the proportion of individuals in the predator population (feeding and non-feeding) observed to be feeding on prey species *x*, and *F_i_* denotes the proportion of the population's feeding individuals observed to be consuming the *i* ^th^ prey species [29]. Species *x* is an arbitrarily chosen species used throughout the calculation of all prey-specific attack rates [29]. Prey-specific feeding rates (*C_i_*, the grams of prey tissue consumed per predator per m^2^ per day) were then calculated based on the multispecies Type II functional response (on which Eqn. 6 is based)
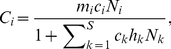
(7)
[cf. 31] where *S* is the total number of prey observed in the predator's diet and *m_i_* is the *i* ^th^ prey's average dry tissue weight. We calculated prey weight from the sizes of prey items observed during the feeding surveys using species-specific allometric relationships [28]. Feeding rates were converted to proportional diet contributions for comparison to those inferred using stable isotope data (Table 1).

#### Stable isotope estimates of prey contributions

Individuals of *H. scobina* (12–15 mm shell length, n = 10) and each of its observed prey (n = 5–8 per species) were collected for stable isotope analysis from both high and mid-intertidal zones in July of 2004 and 2005. All individuals were stored live on ice for 4 hrs and frozen prior to processing. Insufficient material was obtained for *Nr* to enable analysis. Although only a single *Nr* predation event was observed during feeding surveys, we account for this missing prey source by substituting *Nr* with the limpet *Patelloida corticata* (*Pc*), a closely related species that shares the same habitat (Table 2). Analysis of the system without *Pc* did not change the applicability of our weighting method (Fig. S2).

**Table 2:** Mean and standard deviation of species-specific isotopic signatures at Tauranga Head, New Zealand.

Species	Abb.	δ^15^N		δ^13^C		n
		Mean	St.Dev	Mean	St.Dev	
**Whelk Predator**						
*Haustrom scobina*	*Hs*	10.4	0.7	−17.1	0.3	7
**Snails and limpets**						
*Austrolittorina antipodum*	*Aa*	7.6	0.1	−14.8	0.3	5
*Austrolittorina cincta*	*Ac*	8.3	0	−17.8	0.7	2
*Patelloida corticata* *	*Pc*	9.3	0.4	−11.9	0.2	5
*Risellopsis varia*	*Rv*	8.7	0.1	−15.3	0.4	5
**Mussels**						
*Mytilus galloprovincialis*	*Mg*	7.7	0.2	−18.6	0.3	5
*Xenostrobus pulex*	*Xp*	7.9	0.4	−18.8	0.2	5
**Barnacles**						
*Chamaesipho columna*	*Cc*	9.3	0.3	−18.6	0.6	7
*Epopella plicata*	*Ep*	11.6	0.4	−18.7	0.2	7

*Patelloida corticata is a substitute prey for the limpet *Notoacmea* sp. (see text for details).

We dissected, rinsed, and oven-dried the foot muscle tissue of all snails and limpets (*Hs*, *Aa*, *Ac*, *Pc*, *Rv*) and the whole-body tissue of the mussels (*Mg*, *Xp*) and barnacles (*Cc*, *Ep*). The ground samples of some species were pooled to ensure sufficient sample size (Table 2). Analyses of carbon and nitrogen stable isotope ratios were perfformed by the UC Davis Stable Isotopes Facility and were expressed in delta-notation such that δ = 1000((R_sample_/R_standard_)−1) where R = either ^13^C/^12^C or ^15^N/^14^N; reference standards are Vienna PeeDee-belemnite for carbon and atmospheric N_2_ for nitrogen. The isotope values of the predator *Hs* were corrected by −1±0.2‰ and −2±0.3‰ for δ^13^C and δ^15^N, respectively, to account for trophic enrichment (± SD). Chosen values are representative of those observed for invertebrates in general [32].

## Results

### Hypothetical Dataset and Model Assessment

**Figure 1:**
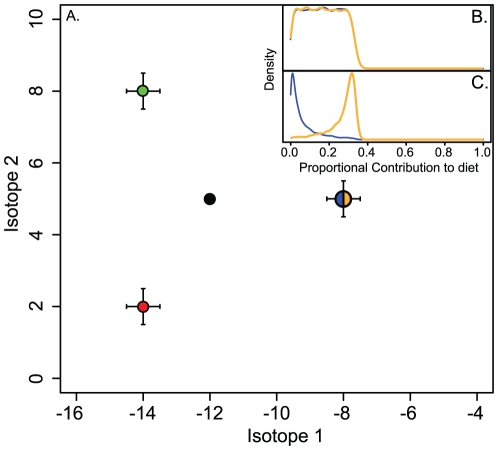
Isotopic niche space of a theoretical single predator, four prey community. **A**) The predator (black) and prey (red, green, blue, orange) have a standard deviation of (0.5, 0.5) for both isotope tracer 1 and 2. Two of the four prey (red and green) are isotopically distinct with no overlap. Two of the four prey (blue and orange) are isotopically identical, with *w_ij_* = 1. **B**) Initial MixSIR contribution-to-diet posterior probability distributions for the isotopically identical prey. **C**) Final weighted and renormalized posterior probability distributions for the isotopically similar prey. Relative abundance values of 0.9 and 0.1 were applied to the orange and blue prey, respectively.

A hypothetical predator-prey isotopic dataset (Fig. 1A) was assigned a single consumer species, and four prey species, each normally distributed across the bivariate isotopic niche space (consumer and prey standard deviation (SD) = 0.5 for each isotopic tracer). Two prey were constructed to have distinct (non-overlapping) isotopic distributions, while the blue and orange prey (hereafter referred to as prey *i* and *j*, respectively) were given equal values. The mixing space was designed such that the overlap of prey *i* and *j* could be manipulated without affecting direct mixing model output (Fig. S3). As expected, analysis of this system with a Bayesian isotope mixing model (MixSIR v.1.0.4) provided similar posterior probability distributions of contribution-to-diet values for the two overlapping prey species (Fig. 1B): proportional contribution medians (1^st^ quartile, 3^rd^ quartile) for prey *i* and *j* = 0.17 (0.08, 0.25), 0.16 (0.08, 0.25). To evaluate isotopic similarity between prey *i* and *j*, we employed Pianka's measure of density overlap, such that *w_ij_* = 1. We then set prey availability values (*α_i_*, *α_j_*) to 0.1 and 0.9 for prey *i* and *j*, respectively, and applied these values to Eqs. 4 and 5 across the entire matrix of iterated diet-to-contribution vectors to calculate the revised estimates of dietary reliance (Fig. 1C): revised proportional contribution medians (1^st^ quartile, 3^rd^ quartile) for prey *i* and *j* = 0.04 (0.01, 0.09), 0.30 (0.25, 0.32).

**Figure 2:**
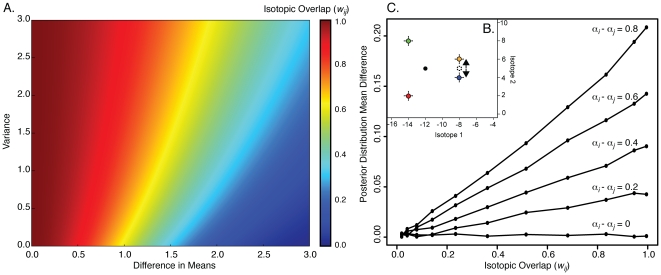
Sensitivity analysis of the weighting procedure as a function of isotopic similarity and the difference in proportional availability of isotopically similar prey. **A**) Sensitivity analysis of Pianka's measure of density overlap (*w_ij_*) across differences in mean values and variance of two bivariate normal isotopic distributions. When variance is small, *w_ij_* decreases sigmoidally as the difference in means increases. Larger variance predictably tends to linearize the relationship. **B**) The mixing space of our hypothetical scenario permits manipulation of isotopic overlap between two prey (blue and orange; the consumer is black) without altering mixing model estimates of summed proportional contribution to diet of the two prey (cf. Fig. S3). **C**) An analysis of the effect of model parameters (density overlap, *w_ij_*, and availability differences, *α_i_*−*α_j_*) on the final weighted and renormalized posterior distributions, measured by the difference of final distribution means for blue and orange in the hypothetical isotopic dataset (Fig. 1). The model is assessed across all possible values of *w_ij_* (0∶1), and across five prey availability scenarios. There is a strong linear effect of isotopic overlap on the difference in means of the weighted posterior probability distributions. The slope of this relationship is determined by availability differences.

An examination of Pianka's measure of overlap confirms consistent behavior over a range of isotopic mean differences and variances, resulting in a sigmoid relationship between the mean difference of prey isotope values and *w_ij_* that becomes less pronounced with increased variance (Fig. 2A). Because we constructed the mixing space such that estimates of dietary contribution are nearly invariant with respect to manipulation of *w_ij_* (cf. Fig. S3), an assessment of the behavior of our weighting procedure across different ratios of prey availability is possible (Fig. 2B,C). There is a linear relationship between the initial degree of isotopic overlap of two prey and the difference of final posterior probability distribution mean values. The slope of this relationship is determined by the availability differences (*α_i_*−*α_j_*); small variations in the linear trends can be attributed to fluctuations in mean values as the distributions are successively weighted and renormalized. If the abundances of prey *i* and *j* are equal (*α_i_*−*α_j_* = 0), our weighting procedure returns results identical to those originally calculated by the isotope mixing model, as intended given its *a priori* ultrageneralist assumption. As the overlap of prey isotope values increases, the weighting procedure increasingly returns dietary contribution values influenced by the relative availability of the two isotopically similar prey. This translates to an increasing discrepancy between the proportional contribution to diet distributions of prey *i* and *j* as the difference in availability increases.

### New Zealand Dataset and Model Validation

**Figure 3:**
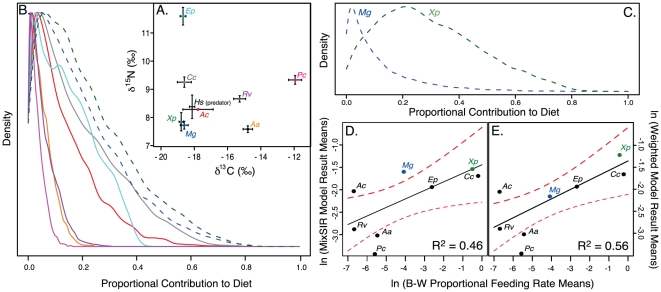
The weighting procedure applied to a New Zealand intertidal community. **A**) Isotopic niche space of the New Zealand whelk-predator system, where δ^13^C is plotted on the x-axis and δ^15^N is plotted on the y-axis. The δ^13^C and δ^15^N values of the predator *Hs* are adjusted for trophic discrimination factors (see methods). **B**) MixSIR contribution-to-diet posterior probability distributions of all New Zealand prey for predator *Hs*. Solid distribution density lines denote isotopically distinct prey, whereas hatched distribution density lines denote isotopically similar prey. Prey colors match those of panel 3A. **C**) Weighted posterior probably distributions for the isotopically similar prey. Relative abundance values of 0.05 and 0.95 correspond to *Mg* (blue) and *Xp* (green), respectively. **D**) ln-ln-transformed regression of biomass-weighted proportional feeding rate means vs. original mixing model result means; (slope = 0.19, and R^2^ = 0.46). Hatched red lines represent the 90% confidence interval. **E**) ln-ln-transformed regression of biomass-weighted proportional feeding rate means vs. weighted model result means; slope = 0.22, R^2^ = 0.56. Hatched red lines represent the 90% confidence interval.

Haustrum scobina's diet at the site includes eight potential prey (*Aa*, *Ac*, *Cc*, *Ep*, *Mg*, *Pc* (substituted for *Nr*), *Rv*, and *Xp*). Five of the eight prey species are isotopically distinct, however the nesting mussel *Xenostrobus pulex* (*Xp*) and the blue mussel *Mytilus galloprovincialis* (*Mg*) had overlapping isotope values; *w_ij_* = 0.63 (Fig. 3A). An analysis of the mixing space reveals a range of likely contributions for each prey (Fig. 3B). The isotopic similarity between *Xp* and *Mg* resulted in similar contribution-to-diet probability distributions for these species in unweighted MixSIR results (Fig. 3B; blue and green hatched distribution density lines, respectively): *Mg* = 0.16 (0.07, 0.28), *Xp* = 0.18 (0.08, 0.31); median (1^st^ quartile, 3^rd^ quartile). Applying relative abundance measurements (*α_Mg_* = 0.05_,_ *α_Xp_* = 0.95) to our weighting procedure resulted in proportional contribution distributions that were quite different (Fig. 3C): *Mg* = 0.07 (0.03, 0.16), *Xp* = 0.27 (0.15, 0.41).

To investigate whether our weighting procedure improved inferences of prey contribution to the diet of *Hs*, we regressed both original mixing model results and our weighted results against *Haustrum scobina*'s field-estimated proportional feeding rates on each of its prey species (Fig. 3D,E). Recall that our field-estimated feeding rates were weighted by prey biomass to ensure a more direct comparison to the isotopic representation of the system, which is intrinsically mass-dependent (Eq. 7). The ln-ln-transformed regression of the mixing model result means with feeding rate observation means (Fig. 3D) indicated that four of the five most heavily preyed upon species (prey with predator feeding rates greater than 0.0023 grams⋅whelk^−1^⋅day^−1^), with the exception of *Mg*, fall within the 90% confidence interval (slope = 0.19, and R^2^ = 0.46). After the implementation of our weighting procedure, all five of the top prey species fall within the 90% confidence interval (Fig. 3E; slope = 0.22, R^2^ = 0.56). Accordingly, the inclusion of prey availability, conditional on the isotopic uncertainty of prey, increased the slope and improved the accuracy of trophic interaction predictions by ca. 10%.

## Discussion

The usefulness of a model is ranked by its ability to accurately describe reality with a minimal number of assumptions [33]. A Bayesian isotope mixing model with an uninformative prior assumes that the consumer is an ultrageneralist until proven otherwise. When multiple prey have similar isotope values, the ultrageneralist assumption becomes realized in the mixing model results, forcing the consumer to have equal contributions of each prey source. Because ultrageneralism is an unrealistic assumption in most cases, we developed a procedure by which different types of biological information can be used to revise this assumption. Our method enables partitioning of prey that are isotopically similar but differ in their relative availability to a predator. We reiterate that prey availability values could represent proportional differences in available biomass, predator preference, relative digestibility, or any (combination of) measurements influencing the availability of prey biomass to a predator. As such, the inclusion of neutral interaction assumptions (consumption in proportion to the prey's abundance) is conditioned on the isotopic uncertainty of prey; the isotope values of prey that are isotopically distinct remain dominantly informative.

Our isotopic analysis of a New Zealand intertidal system, compared to field-measured feeding rates, reveals increased accuracy in predicted consumption rates of *Haustrum scobina* on its prey after the weighting procedure was employed (Fig. 3D,E). Neutral interaction assumptions were incorporated only for isotopically similar prey (the mussels *Xenostrobus pilex* and *Mytilus galloprovincialis*), thereby avoiding the incorporation of such assumptions for all prey. Our results suggest that, in this case, we interchange the formalism of the Bayesian paradigm with predictive power, thereby validating the use of such methods in cases where neutral interaction assumptions cannot be justified, or when appropriate prior probability distributions cannot be constructed, for all potential prey.

The ultimate aim of our method is to increase the accuracy of trophic interaction estimates, thereby contributing to our understanding of ecological systems, the role of neutral and niche processes in organizing communities, and the structure of food webs. Here we address the issue of isotopic similarity concerning a pair of prey, however in more complex scenarios, multiple iterations of this method could be carried out for multiple pairs of overlapping prey. In principle, it would also be possible to handle larger numbers of overlapping prey within the framework that we have presented, and this would be a useful future addition to this work. Whether there are few or many isotopically overlapping resources, the ability of our method to refine estimates of prey contribution to a consumer's diet relies on the accurate incorporation of proportional prey availability data. Although the availability of a prey may be the result of differential abundance (emphasized here), it may also result from consumer behavior, where certain prey are preferred over others. As long as prey availability can be expressed as proportions (where the proportional availability sums to unity over the isotopically overlapping prey), such data can be used in a manner equivalent to our example of differential prey abundance. The accuracy of our method is entirely dependent on the accuracy of proportional availability measurements. If prey availability data are highly uncertain or unknown for one or more of the isotopically overlapping prey sources, our method is of little utility. Furthermore, if multiple mechanisms are important in constraining prey availability, the relative influence of each mechanism must be known such that the ‘overall’ proportional availability of a given prey can be estimated.

There are situations when it is difficult or impossible to inform Bayesian isotope mixing models with availability data. Such issues contribute to difficulties in developing suitable priors for prey species in many systems, though an exploration of prior development in the context of consumer-resource relationships, particularly for isotope mixing models, deserves additional investigation. Formulation of prior distributions based on availability data may be possible and appropriate in systems that are observable and where neutral interaction assumptions can be justified. However, ratios of stable isotopes are often considered to be of greatest utility when used to investigate systems that are difficult, if not impossible, to observe directly [4], [6], [19]. Our procedure enables investigators to employ independent biological information to gain additional insights from isotopic data, while simultaneously relaxing the number of required parameters relative to traditional Bayesian models of consumer resource use.

## Supporting Information

Figure S1 **Species accumulation curve (± SD) of the prey observed in the diet of** ***Haustrum scobina*** **at Tauranga Head, constructed using feeding survey observations as the unit of sampling** [34]. Gotelli N.J. & Colwell R.K. (2001). Quantifying biodiversity: procedures and pitfalls in the measurement and comparison of species richness. *Ecol. Lett.*, 4, 379–391.
(EPS)

Figure S2 **An analysis of the New Zealand system excluding the prey** ***Patelloida corticata*** **(** ***Pc*** **).** As expected, there are slight differences in original MixSIR results, however, applying prey availability data to the overlapping prey *Mg* and *Xp* results in a similar increase in the accuracy of prey contribution estimations. **A**) ln-ln-transformed regression of biomass-weighted proportional feeding rate means vs. original mixing model result means; slope = 0.08, R^2^ = 0.42. **B**) ln-ln-transformed regression of biomass-weighted proportional feeding rate means vs. weighted model result means; slope = 0.11, R^2^ = 0.53. The hatched red lines represent the 90% confidence interval.
(EPS)

Figure S3 **Manipulation of the isotopic overlap of prey.** **A**) A mixing space with 2 non-overlapping prey (green, red), two overlapping prey (blue, orange), and a single consumer (black). Here, the overlap of blue and orange prey (*w_blue,orange_*) = 1. **B**) MixSIR estimates of % contribution to diet for each prey associated with Fig. S.3.A. Green and red are predicted to contribute equally to the consumer's diet, as are blue and orange. **C**) An alternative mixing space such *w_blue,orange_* = 0. Here, the blue and orange prey have been symmetrically moved from their previous isotopic values (δ^13^C = −8, δ^15^N = 5) to Blue: δ^13^C = −8, δ^15^N = 4; Orange: δ^13^C = −8, δ^15^N = 6. **D**) MixSIR estimates of % contribution to diet for each prey associated with Fig. S.3.C. Although the overlap of blue and orange has been manipulated, the geometry of the mixing space retains similar estimates of % contribution to diet for all prey, with only slight differences in variance.
(EPS)
